# Sciatica

**Published:** 1844-12

**Authors:** 


*Sciatica.*—Painful affections of this nerve, though classed under the same name, are very various.

Acute inflammation of the nerve is most frequently the effect of exposure to wet and cold, standing or sitting in water, on wet ground, in boats or coaches. The pain in the course of the nerve is very severe, aggravated by the slightest motion, and attended by high febrile symptoms. It requires strictly antiphlogistic treatment. Cupping along the course of the nerve, brisk purging with calomel, followed by salts and senna, each dose containing thirty drops of vin. colchici, or three to six grains of powdered colchicum, every five hours, until the febrile symptoms are diminished. Then ten to fifteen grains of Dover's powder will greatly relieve the pain; for the pain is seldom reduced in proportion to the reduction of the febrile symptoms, a point which should be remembered. At this stage two grains of calomel, from four to six of powdered colchicum, and five of Dover's powder, should be given every six hours, with some diuretic, and an extra dose of opium at night, if necessary; repeating the black draught every second morning. It is needless to add that the effect of the colchicum should be watched. Even after these means, the pain may be obstinate and severe, requiring blistering. It may be necessary to continue the calomel until the gums are sore, as it is important to prevent the disease from becoming chronic, which it is very prone to do.

This chronic inflammation of the nerve is obstinate, and sometimes followed by loss of power, wasting and shrinking of the limb. The pain does not wholly subside, but is aggravated in paroxysms, and particularly at night. In some cases there is nocturnal fever. To distinguish this state from one of pure neuralgia, is often difficult. Nocturnal fever, and scanty high-colored urine with sediment, sometimes assists the diagnosis. If thé patient has this chronic form of the disease, when first seen, purgatives should be given, especially if his age is advanced,. Calomel and colocynth, followed by castor oil, sometimes bring away hardened fæces, and occasionally perseverance in the purgatives has relieved the disease, which seemed to depend on a loaded state of the bowels; but in general mercury will be necessary. The following formula Dr. Hunt, as he says in his late work, on Tic Douloureux, &c., employs:—R. Hydrargyri phosphatis, gr. j.; opii., gr. j.; antim. potassæ tart. gr. 1-6. Fiat pilula omni nocte sumenda.

If there is much nocturnal fever, in addition to this, moderate doses of nitrate of potash and colchicum should be given three times a day, with occasional aperients. If much exhaustion of strength and emaciation, the compound decoction of sarsaparilla with the liquid extract during the day, and the mercurial pill at night. Counter irritation is especially useful. Open blisters, the tartar-emetic plaster, or even a caustic issue behind the trochanter. Dr. Hunt alludes to the hydriodate of potash as producing a powerful effect in some cases, but he says he has not himself used it.

Sciatica, of which pain is the only symptom, returning in paroxysms like electric shocks, is occasionally met with, and should be treated like pure neuralgia, first by purgatives, and then by steel, quinine or arsenic with sedatives.

A case is given in which sciatica existed with piles and prolapsus ani, and the removal of the piles relieved the pain.

Persons subject to sciatica should wear flannel drawers next to the skin, and if they are very susceptible to weather, washed-leather drawers over the flannel, during the winter months. We have seen benefit in some cases of obstinate pain in the sciatic nerve from washed-leather drawers worn next to the skin.—*Brit. and For. Med. Rev.*

